# Femoral components are positioned in greater external rotation using functional alignment in robot‐assisted total knee arthroplasty compared to mechanical alignment

**DOI:** 10.1002/jeo2.70362

**Published:** 2025-07-21

**Authors:** Anne Ruth van Meijeren, Danielle Langeloo, Hans‐Peter van Jonbergen, Marrigje Meijer

**Affiliations:** ^1^ Department of Orthopedic Surgery Deventer Hospital Deventer The Netherlands

**Keywords:** functional alignment, mechanical alignment, robotic‐assisted total knee arthroplasty, soft tissue balance, total knee arthroplasty

## Abstract

**Purpose:**

Objective of this study was to evaluate the effect of functional alignment on femoral component rotation compared to mechanical alignment in knees with a constitutional tibial varus alignment classified as coronal plane alignment of the knee (CPAK) types I, II and IV.

**Methods:**

This retrospective study included patients undergoing conventional total knee replacement (TKR) (*n* = 64) and robot‐assisted TKR (*n* = 84). Coronal and axial measurements were performed manually and automatically using pre‐ and postoperative computed tomography images in the conventional group and robot‐assisted group respectively.

**Results:**

Femoral component rotation was statistically significant more external rotated in the conventional group versus robot‐assisted group (1.3° ± 2.7° vs. 0.76° ± 1.4°, *p* < 0.001). Also, the tibial plateau was placed statistically significant more in varus in the robot‐assisted group compared to the conventional group (87.6° ± 2.5° vs. 88.9° ± 1.4°, *p* < 0.001).

**Conclusion:**

Functional alignment leads to more varus of the tibial component and less external rotation of the femoral component compared to mechanical alignment in TKR in patients with a constitutional tibial varus alignment. Since this is a radiological analysis, further research is needed to understand how these differences affect clinical outcomes as faster recovery, adequate patellar tracking and longer survival.

**Level of Evidence:**

Level III.

**Abbreviations**
ASA: American Society of Anesthesiologists
BMI: body mass index
CPAK: coronal plane alignment of the knee
CT: computed tomography
FA: functional alignment
HKA: hip‐knee‐ankle angle
JLO: joint line obliquity
LLR: long leg radiograph
MA: mechanical alignment
mLDFA: mechanical lateral distal femoral angle
mMPTA: mechanical medial proximal tibial angle
SD: standard deviation
SPSS: Statistical Package for the Social Sciences
sTEA: surgical transepicondylar axis
TKR: total knee replacement

## INTRODUCTION

Despite advances in total knee replacement (TKR), approximately 10% of patients remain dissatisfied with their outcomes [6]. Up to this date, the underlying reason is unclear. This ongoing dissatisfaction has led to more interest in how different alignment techniques affect the position of the implant and clinical outcomes.

Proper alignment and ligament balancing during TKR are essential for both good clinical outcome and prosthesis survival [9, 21]. Mechanical alignment (MA), generally considered, is the reference standard for TKR component positioning [10]. This technique aims to position the joint line, and thus the components, perpendicular to the mechanical axis in the coronal plane to minimise implant wear. In MA, the native anatomy of an individual patient is not considered. Tibial alignment is especially important because it affects load distribution and whether ligament balancing is needed.

Macdessi et al. described the Coronal Plane Alignment of the Knee (CPAK), a classification system that identifies nine distinct types of coronal lower limb alignment, which accounts for native coronal alignment and joint line obliquity [15]. This classification can be used to analyse which knee types might benefit from ligament balancing during TKR [15]. The most common native knee alignment is tibial varus. CPAK types I, II and IV represent this constitutional varus pattern, found in knees with > 3° tibial varus. Together, they account for the majority of lower leg alignment types with a total of 62.3% of patients with osteo‐arthritis (Type I: 18.8%, Type II: 33.5%, Type IV: 10.0%) [15, 20, 22].

Conventional TKR using MA in patients with a constitutional varus requires medial soft tissue release to achieve a ligament‐balanced knee in the coronal plane. MA will also result in an asymmetrical flexion gap. To compensate for this asymmetrical flexion gap, external rotation of the femoral component is needed. However, this approach might potentially result in adverse issues, such as patellofemoral maltracking, instability and pain ultimately leading to suboptimal postoperative clinical outcomes [7, 23].

Functional alignment (FA) is a novel strategy, which might be more suitable to achieve optimal ligament balancing in these cases. This technique requires use of a robot and aims to restore native alignment of the joint, directly resulting in a balanced knee [4]. In theory, no adjustments need to be made in femoral rotation to compensate for an asymmetrical flexion gap. As a result, the femoral component is positioned more neutrally in the axial plane [24].

Our hypothesis is that the use of FA strategy results in less external rotation of the femoral component compared to MA strategy. The primary aim of this retrospective study is to assess the femoral and tibial angles in the coronal and axial planes in patients with a constitutional tibial varus alignment.

## MATERIALS AND METHODS

Between November 2021 and November 2024, we recruited 203 patients with primary symptomatic osteoarthritis of the knee who were eligible for TKR at the Deventer Hospital. Institutional review board agreement was obtained for this study. All individual participants provided written informed consent. For both groups, only patients with a constitutional tibial varus alignment classified as CPAK I, II or IV were included. Although we did not directly compare CPAK subtypes, the classification was used to ensure that all included patients had a similar coronal alignment pattern, allowing for a more consistent comparison between alignment techniques.

This retrospective cohort study compared TKR procedures using either MA or FA technique. FA procedures were performed using the MAKO robotic‐arm system (Stryker, Kalamazoo, MI, USA), while MA procedures were performed with conventional instrumentation using the Persona Knee System (Zimmer Biomet, Warsaw, IN, USA). Included were patients with a primary diagnosis of symptomatic osteoarthritis eligible for TKA. Excluded were patients who underwent revision surgery or used revision components, or those who received any prosthesis other than the Persona Knee system for the conventional TKR group or the Triathlon CR prosthesis for the robot‐assisted TKR group. Furthermore, individuals with limited knowledge of the Dutch language or mental incapacity to participate in the study were excluded.

For the primary study aim, we defined two groups. Group A consisted of 64 patients that had conventional TKR with MA. Group B consisted of 84 patients that had robot‐assisted TKR with FA. All procedures were performed during a transition phase from conventional to robot‐assisted TKR. To minimise bias related to the surgical learning curve, the first 15 cases per surgeon following the introduction of the robotic system were excluded from the analysis.

### Surgical technique

Surgery was performed by three orthopaedic surgeons who have extensive experience with the use of both the conventional and robot‐assisted TKR technique using standard medial arthrotomy.

Conventional TKR (MA strategy) was performed using the Persona PS system. An extramedullary guide was used for tibial resection. Proper rotation of the tibial component was determined using a combination of landmarks including the anterior cortex of the tibia, tibial tuberosity and Akagi's line. For femoral component alignment, an intramedullary guide was used, followed by resection of the distal femur in 5° valgus and external rotation of 3° using the posterior condyles as reference point.

Robot‐assisted TKR (FA strategy) was performed using the MAKO total knee robot arm‐assisted surgery system. Using a 3D‐based model based on preoperative CT, planning and position of femoral and tibial component size was performed. Varus and valgus stress were applied at 0° and 90° knee flexion to assess soft tissue balancing medially and laterally. Functional alignment planning was performed as follows: first the extension gap was made rectangular with adjustment of the coronal angle of the tibia, depending on native joint anatomy and intraoperative ligament balancing under varus and valgus stress testing, followed by balancing the flexion gap. The coronal resection angle was limited to a maximum of 3° varus relative to the mechanical axis. Adjustments were made by adjusting the femoral component rotation. Matching the extension and flexion gap was performed by adjusting the femoral and tibial component levels and adjusting the sagittal slope.

### Radiographic evaluation

For the conventional TKR group pre‐ and postoperative coronal alignment measurements were performed on the CT scout, serving as a non‐weight bearing long leg radiograph (LLR). Postoperative measurement of femoral component rotation was performed on postoperative CT scans according to the Berger protocol [2]. Measurements were done manually by two independent observers.

In the robot‐assisted TKR group, preoperative coronal measurements were performed on CT scan with the MAKO software. Postoperative measurement of femoral component rotation as well as coronal measurement of the tibial component were performed intra‐operatively with the MAKO software using the same anatomical landmarks compared to the conventional TKR group.

**Figure 1:**
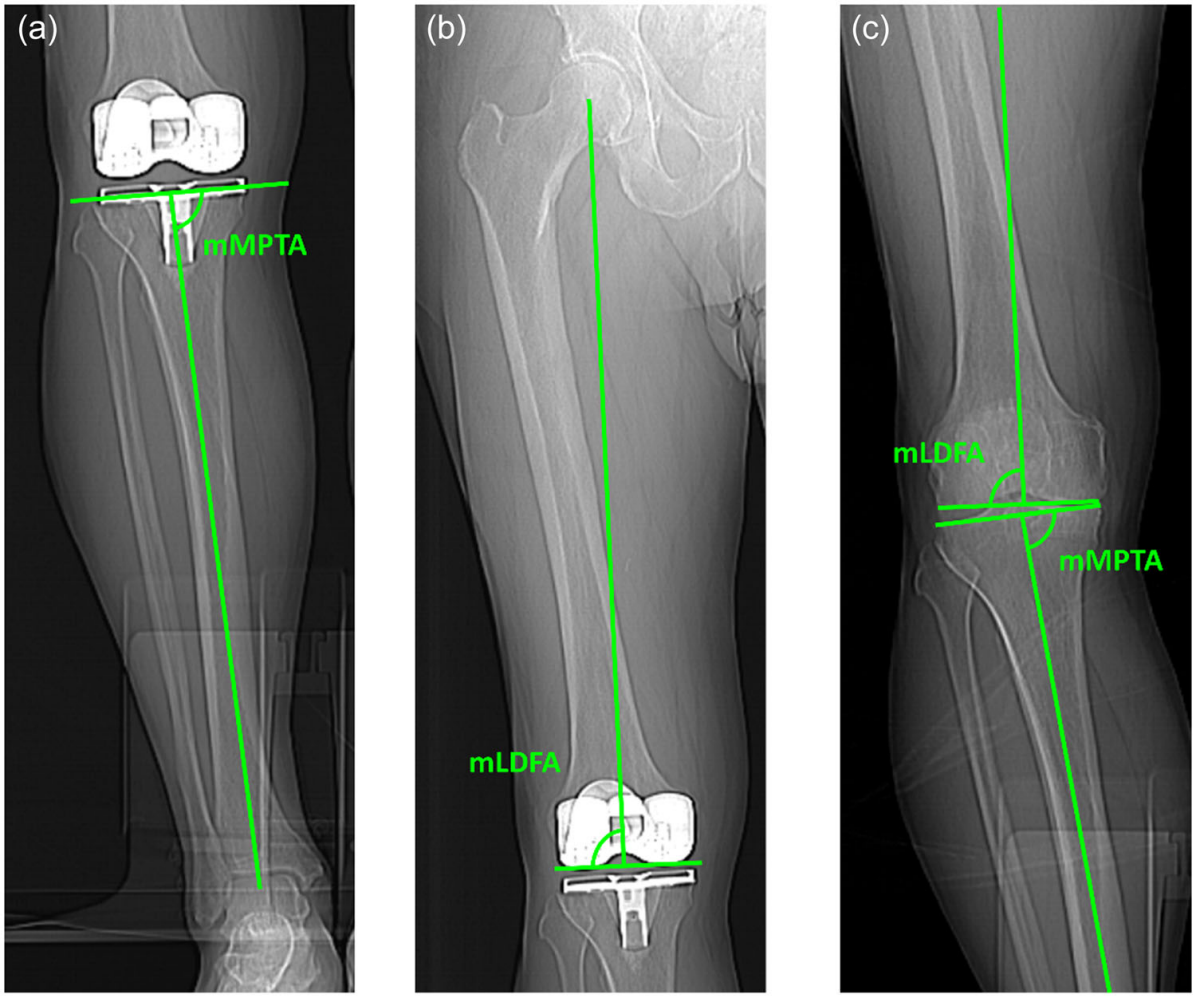
CT femoral and tibial alignment measurement in the coronal plane. CT, computed tomography; mLDFA, mechanical lateral distal femoral angle; mMPTA, mechanical medial proximal tibial angle.

**Figure 2:**
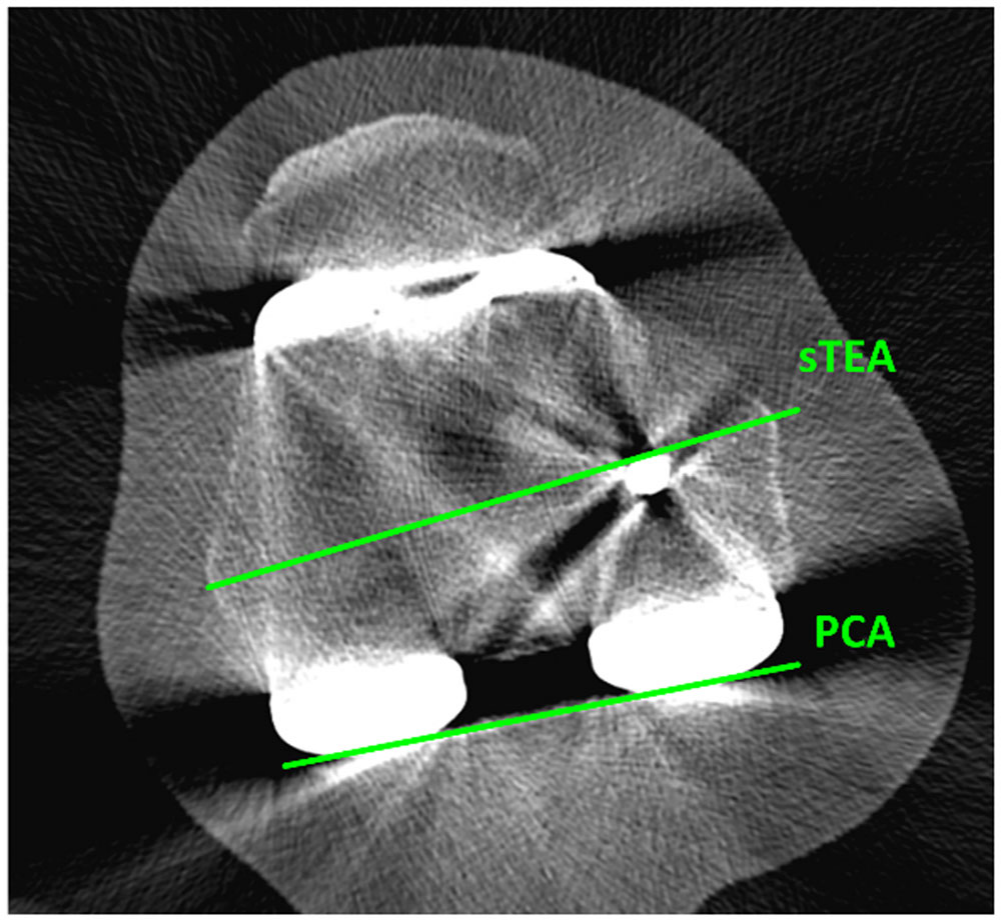
CT femoral component rotation measurement in the axial plane. CT, computed tomography; sTEA, surgical transepicondylar axis.

Angles measured for this study were as follows: the mechanical medial proximal tibial angle (mMPTA) was defined as the angle between the mechanical axis of the tibia and the articular surface of the tibial plateau in the coronal plane on the medial side (Figure 1a,c). The mechanical lateral distal‐femoral angle (mLDFA) was defined as the angle between the mechanical axis of the femur and the line between the most distal contours of the medial and distal femur condyles in the coronal plane on the lateral side (Figure 1b,c). The femoral component rotation angle was defined as the angle between the surgical transepicondylar axis (sTEA) and the prosthetic posterior condylar axis (inner border of posterior cut) (Figure 2). External rotation of the component is shown as a positive (+) angle and internal rotation of the component is shown as a negative (−) angle.

CPAK classification was based on joint line obliquity (JLO) and HKA. JLO was derived by the sum of mMPTA and mLDFA. JLO > 183° indicates a proximal apex joint line, <177° a distal apex joint line, any values in between indicates a neutral joint line [5]. The hip‐knee‐ankle ankle (HKA) was defined as the angle between the femoral and tibial mechanical axes. HKA was considered varus if less than −2°, neutral within −2° to 2° and valgus greater than 2°. All data were stored in SPSS.

### Statistical analysis

Statistical analyses were conducted with Statistical Package for the Social Sciences (SPSS, version 28.0.1.0 for Windows). Baseline characteristics were analysed using descriptive statistics with mean ± standard deviation (SD) for continuous variables and frequencies with respective percentages for discrete and dichotomous variables. Mean and median differences in baseline characteristics and femoral rotation between the conventional TKR group and robot‐assisted TKR group were compared using T‐tests or ANOVA‐tests depending on distribution. For differences in CPAK classification (categorical data), the Kruskal–Wallis test was used. In order to evaluate the effect of tibial component rotation on femoral component rotation, a paired t‐test was used. According to Benjamin et al. a *p*‐Value < 0.005 was considered statistically significant [1].

## RESULTS

Between November 2021 and November 2024, a total of 148 patients were included. The study population was composed of 74 (50%) women and 74 (50%) men. Mean age was 71.7 ± 8.3 years. Conventional TKR was performed on 64 patients and robot‐assisted TKR was performed on 84 patients.

All anthropometric measurements including age, sex, ASA‐classification (American Society of Anesthesiologists), laterality and BMI (body mass index) were comparable between the conventional TKR group and the robot‐assisted TKR group (Table 1). Preoperative HKA, JLO, mMPTA and mLDFA angles and distribution in CPAK‐classification were comparable between the two groups (Table 2). Median mMPTA was 85.5° (IQR 84.0°–86.9°) and median mLDFA was 87.8° (IQR 87.0°–89.9°).

**Table 1:** Group‐specific baseline characteristics.

	Total (*n* = 148)	Conventional TKR (*n* = 64)	Robot‐assisted TKR (*n* = 84)	*p* value
Mean age (years, SD)	71.2 (±8.3)	71.3 (±8.3)	72.1 (±8.2)	0.285
Sex (*n*, %)				0.020
Female	74 (50%)	25 (39%)	49 (58%)	
Male	74 (50%)	39 (61%)	35 (42%)	
BMI (kg/m^2^, SD)	30.1 (±5.0)	30.4 (±4.7)	29.7 (±5.4)	0.183
Side (*n*, %)				0.607
Right	82 (55.4%)	37 (57.8%)	45 (53.6%)	
Left	66 (44.6%)	27 (42.2%)	39 (46.4%)	
ASA‐score (*n*, %)				0.518
I	17 (11.5%)	9 (14.1%)	8 (9.5%)	
II	89 (60.1%)	38 (59.4%)	51 (60.7%)	
III	41 (27.7%)	16 (25.0%)	25 (29.8%)	
IV	1 (0.7%)	1 (1.6%)	0 (0.0%)	

Abbreviations: ASA, American Society of Anesthesiologists; BMI, body mass index; SD, standard deviation; TKR, total knee replacement.

**Table 2:** Preoperative HKA, JLO, mMPTA and mLDFA angles and distribution in CPAK‐classification.

	Total (*n* = 148)	Conventional TKR (*n* = 64)	Robot‐assisted TKR (*n* = 84)	*p* value
CPAK (*n*, %)				0.992
I	71 (48.0%)	31 (48.4%)	40 (47.6%)	
II	58 (39.2%)	25 (39.1%)	33 (39.3%)	
IV	19 (12.8%)	8 (12.5%)	11 (13.1%)	
HKA (°, SD)	−2.8 (±2.9)	−3.2 (±3.3)	−2.6 (±2.5)	0.076
mMPTA (°, SD)	85.4 (±2.1)	85.2 (±2.3)	85.5 (±2.0)	0.216
mLDFA (°, SD)	88.2 (±2.2)	88.5 (±2.3)	88.1 (±2.2)	0.133
JLO (°, SD)	173.6 (±3.3)	173.7 (±3.2)	173.6 (±3.4)	0.414

Abbreviations: CPAK, coronal plane alignment of the knee; HKA, hip‐knee‐ankle angle; JLO, joint line obliquity; mLDFA, mechanical lateral distal femoral angle; mMPTA, mechanical medial proximal tibial angle; SD, standard deviation; TKR, total knee replacement.

The femoral component was significantly more externally rotated postoperatively in the conventional TKR group versus robot‐assisted TKR group (1.3° ± 2.7° vs. 0.8° ± 1.4° respectively, *p* < 0.001). Furthermore, the postoperative mMPTA angle was higher in the conventional TKR group compared to robot‐assisted TKR group (88.9° ± 1.4° vs. 87.6° ± 2.5°, *p* < 0.001) (88.9° vs. 87.6°, *p* < 0.001) (Table 3). In other words, the tibial component was positioned statistically significant more in varus in the robot‐assisted TKR group.

**Table 3:** Comparison rotation of the femoral component and mMPTA of the tibial component between the conventional and robot‐assisted TKR group.

	Total (*n* = 148)	Conventional TKR (*n* = 64)	Robot‐assisted TKR (*n* = 84)	*p* value
Femoral component rotation angle (°, SD)	1.0 (±2.1)	1.3 (±2.7)	0.8 (±1.4)	**<0.001**
Postoperative mMPTA (°, SD)	88.1 (±2.2)	88.9 (±1.4)	87.6 (±2.5)	**<0.001**

Abbreviations: mMPTA, mechanical medial proximal tibial angle; SD, standard deviation; TKR, total knee replacement.

## DISCUSSION

The most important finding of this study was that the MA strategy in patients with a constitutional tibial varus results in a statistically significant more externally rotated femoral component compared to the FA strategy. Also, the tibial component was placed in statistically significant more varus using the FA strategy. Our findings indicate that the use of anatomical landmarks in MA strategy combined with the standard 3° external rotation may not fully account for individual joint line orientation, particularly in varus knees. This might contribute to an overcompensation in femoral rotation to achieve a balanced flexion gap. In contrast, FA aims to respect native joint anatomy and ligament tension for implant positioning, potentially resulting in a more anatomical alignment, reflecting patient's native joint line. The increased external rotation observed in MA likely reflects the need to correct the asymmetrical flexion gap caused by standardised component positioning in varus knees [15, 21]. Since FA allows for intraoperative assessment and adjustments of the flexion and extension gaps, it may reduce the need for such rotational adjustments [11]. These differences in alignment approach may explain the variation in component orientation between the two strategies, particularly in patients with constitutional varus alignment.

Up to this date, there is no consensus regarding which alignment strategy is superior. MA, considered as the reference standard, aims for a neutral leg axis (hip‐knee‐angle (HKA) of 180°) where both femoral and tibial components are placed perpendicular to the mechanical axis [10, 19]. To correct for imbalance in the coronal plane, a medial release is often performed in case of a constitutional tibial varus alignment. The flexion gap is corrected for by externally rotating the femoral component. In contrast to MA, the primary aim of FA is to position TKR components according to the native anatomy and therefore minimising any need for soft‐tissues in the coronal plane [13]. While the difference in femoral external rotation (0.5°) is relatively small, even minor differences can impact patellar tracking and knee kinematics. This difference supports the theory that femoral rotation is influenced by tibial alignment as described earlier. The small difference in femoral rotation is likely a reflection of the limited anatomical variation within our selected patient cohort (CPAK types I, II and IV). Although the clinical relevance of this finding remains uncertain and warrants further investigation, it underlines the need for a more individualised approach to femoral component rotation, rather than a routinely application of external rotation of the femoral component.

Several studies show that patients who undergo robot‐assisted TKR experience a reduced length of hospital stay and improved physical function when compared to those undergoing conventional TKR within a timeframe of one to two years following surgery [5, 16, 17, 18]. Cool et al. found that robot‐assisted TKR leaded to lower 90‐day episode‐of‐care costs compared to conventional TKR due to fewer readmissions and more patients that were discharged home [5]. Other studies demonstrated improved postoperative outcomes after one and two years, including pain, physical function and aseptic failure rates [17, 18]. Interestingly, Kim et al. stated that at a minimum follow‐up period of 10 years showed no differences between robot‐assisted and conventional TKR in terms of functional outcome scores, complications and aseptic loosening in robot‐assisted TKR [12]. However, no differences in implant alignment were found between the conventional and robot‐assisted TKR group. Also, outcomes were not specified for different types of native anatomy (CPAK classification). For different CPAK types, a different alignment strategy might be superior.

Our study has some limitations. First of all, no clinical outcomes and joint gap measurements were included, which limits our ability to confirm whether a truly balanced soft tissue envelope was achieved without ligament releases. Future studies should include these parameters to assess the functional implications of our radiological findings. Second, different measurement techniques were used for coronal and axial component alignment. However, given the high reliability and validity of CT‐based and intraoperative robot‐assisted measurements, the impact on our findings is expected to be minimal [3, 8, 14]. Third, the retrospective study design was due to the transition from conventional to robot‐assisted TKR in our hospital, limiting prospective inclusion to the later cases. Despite this, the design remains appropriate for evaluating femoral component rotation in constitutional tibial varus. Lastly, measurements in the conventional TKR group were performed by two observers, but intra‐ and interrater reliability is high to excellent as noted earlier.

The results of this study are of clinical relevance as they raise concerns regarding the MA strategy. As MA does not take into account native anatomy, additional soft‐tissue releases have to be performed and the femoral component needs to be externally rotated additionally to achieve ligament balancing. In FA, where native anatomy is leading, additional external rotation and soft‐tissue releases are not needed to achieve a ligament balanced total knee prosthesis.

## CONCLUSION

MA leads to a more externally rotated femoral component compared to FA in TKR in patients with a constitutional tibial varus alignment. When following the native anatomy during TKR, the femoral component can be aligned more naturally and therefore the flexion gap is automatically balanced. External rotation of the femoral component may not be necessary. This might result in better clinical results and survival when using de FA strategy. Further studies are needed to evaluate whether the FA strategy leads to improved long‐term clinical outcomes and prosthesis survival.

## AUTHOR CONTRIBUTIONS

Anne Ruth van Meijeren and Marrigje Meijer performed the measurements. Anne Ruth van Meijeren, Danielle Langeloo, Hans‐Peter van Jonbergen, and Marrigje Meijer were involved in study planning and supervision. Anne Ruth van Meijeren processed the data, performed the analysis, designed the figures, and drafted the manuscript. Danielle Langeloo, Hans‐Peter van Jonbergen, and Marrigje Meijer provided substantial feedback and edits to the final version. All authors contributed to the interpretation of the results and were involved in critical revision of the manuscript.

## CONFLICT OF INTEREST STATEMENT

The authors declare no conflict of interest.

## ETHICS STATEMENT

Institutional Review Board Deventer Ziekenhuis reference number ME24‐57. Informed consent was obtained from all patients to use their data.

## Data Availability

None declared.
